# Overcoming resistance by ALK compound mutation (I1171S + G1269A) after sequential treatment of multiple ALK inhibitors in non‐small cell lung cancer

**DOI:** 10.1111/1759-7714.13299

**Published:** 2020-01-13

**Authors:** Ken Takahashi, Yosuke Seto, Koutaroh Okada, Shinya Uematsu, Ken Uchibori, Mika Tsukahara, Tomoko Oh‐hara, Naoya Fujita, Noriko Yanagitani, Makoto Nishio, Kenichi Okubo, Ryohei Katayama

**Affiliations:** ^1^ Division of Experimental Chemotherapy, Cancer Chemotherapy Center Japanese Foundation for Cancer Research Tokyo Japan; ^2^ Department of Thoracic Surgery Tokyo Medical and Dental University Tokyo Japan; ^3^ Department of Thoracic Medical Oncology The Cancer Institute Hospital, Japanese Foundation for Cancer Research Tokyo Japan; ^4^ Cancer Chemotherapy Center, Japanese Foundation for Cancer Research Tokyo Japan

**Keywords:** ALK compound mutation, next‐generation sequence, non‐small cell lung cancer

## Abstract

**Background:**

Anaplastic lymphoma kinase (ALK) fusion genes are found in 3%–5% of non‐small cell lung cancers (NSCLCs). ALK inhibitors show a very high response rate to ALK‐positive NSCLCs. However, the emergence of acquired resistance is inevitable. In this study, we investigated the drugs for overcoming resistance especially compound mutations after sequential treatment with crizotinib, alectinib, and lorlatinib.

**Method:**

Next‐generation sequencing (NGS) and Sanger sequencing were performed on a liver biopsy tissue obtained from a clinical case. Ba/F3 cells in which mutant EML4‐ALK were overexpressed were prepared, and cell viability assay and immunoblotting were performed to check the sensitivity of five independent ALK inhibitors.

**Results:**

I1171S + G1269A double mutation was identified by NGS and Sanger sequencing on a liver biopsy tissue from a patient who relapsed on lorlatinib treatment. Ceritinib and brigatinib—but not other ALK inhibitors—were active against the compound mutations in the cell line model.

**Conclusions:**

With the sequential ALK inhibitors treatment, cancer cells accumulate new mutations in addition to mutations acquired previously. The identified compound mutation (I1171S + G1269A) was found to be sensitive to ceritinib and brigatinib, and indeed the patient's tumor partially responded to ceritinib.

**Key points:**

- ALK compound mutation was found in a clinical sample that was resistant to lorlatinib after sequential ALK‐tyrosine kinase inhibitor (TKI) treatment.
- Ceritinib and brigatinib are potential overcoming drugs against ALK I1171S + G1269A double mutation.

## Introduction

Anaplastic lymphoma kinase (ALK) fusion genes are found in 3%–5% of all non‐small cell lung cancers (NSCLCs).^1^ ALK fusion protein constitutively activates ALK‐tyrosine kinase, which increases cell proliferation and becomes cancerous. ALK inhibitors bind to ALK‐tyrosine kinase and show a high response rate to ALK‐rearranged lung cancer.^2^

Previously, the first‐generation ALK‐tyrosine kinase inhibitor (TKI) crizotinib was the standard treatment for newly diagnosed cases of ALK rearrangement NSCLC, because crizotinib showed a response rate of more than 60% and a median progression‐free survival (PFS) of approximately one year in clinical trials.^3^ However, there was a problem because resistance developed within a couple of years after the treatment started in most of the patients. The major resistance mechanisms to crizotinib included mutations in ALK‐tyrosine kinase domains such as L1196M, G1269A, and C1156Y. Alectinib and ceritinib as second‐generation ALK‐TKIs have been shown to be effective in crizotinib‐resistant patients with secondary mutations except for a few resistance mutations such as G1202R.^4^ Since the direct comparison of crizotinib and alectinib as first‐line therapy in two phase III clinical trials, alectinib has provided significantly longer PFS (more than 34 months in alectinib vs. less than 12 months in crizotinib), and is now widely used as the first‐line therapy.^5^, ^6^ Gainor *et al*. reported that approximately 20%–30% of G1202R mutations occur after treatment with second‐generation ALK inhibitors, and the third‐generation ALK inhibitor lorlatinib is active against their acquired resistance.^4^ Thus, patients with ALK‐rearranged NSCLC usually receive the sequential ALK‐TKI treatment (previously crizotinib followed by alectinib or ceritinib; currently, alectinib followed by other ALK‐TKIs, e.g., ceritinib, lorlatinib, brigatinib [not in Japan], or crizotinib). However, in several reports, diverse compound ALK mutations were identified in lorlatinib‐resistant cells or patient samples after these patients had received the sequential ALK‐TKI treatments.^7^, ^8^, ^9^ The previously reported compound mutations found in patients are F1174V + G1202R,^10^ C1156Y + L1198F,^7^ E1210K + S1206C, E1210K + D1203N,^4^ C1156Y + L1198F, I1171N + L1198F, L1171N + D1203N, L1196M + G1202R, G1202R + G1269A, D1203N + E1210K, G1202R + L1204V + G1269A, D1203N + E1210K + G1269A,^8^ R1192P + G1202R,^11^ G1202R + G1269A,^12^ F1174L + G1202R, T1151M + G1202R,^13^ L1196M + G1202R, I1171N + D1203N.^14^ Therefore, the search for drugs that can overcome these compound mutations is crucial and urgent. Several reports have shown that some of the lorlatinib‐resistant compound mutations became resensitized to first‐ or second‐generation ALK inhibitors (e.g., C1156Y + L1198F became resensitized to crizotinib, and I1171N + L1256F became resensitized to alectinib).^7^, ^9^

In this report, we describe a case in which I1171S + G1269A compound mutation was acquired after exposure to crizotinib, alectinib, and lorlatinib. As there was no available information about the drug sensitivity of this compound mutation, we prepared cell lines in which EML4‐ALK wild‐type (WT) and mutants were overexpressed; cell viability assay and immunoblotting analysis were subsequently performed.

## Methods

### Clinical sample

The patient provided informed consent for all genetic and cell biological analyses which were performed in accordance with the protocols approved by the Institutional Review Board of the Japanese Foundation for Cancer Research, the Ethics Committee.

### Polymerase chain reaction amplification and Sanger sequencing

Total RNA was extracted from a specimen of clinical liver metastasis (JFCR 49‐2) using Maxwell RSC simplyRNA Tissue Kits (Promega Corp., Madison, WI, USA) according to the manufacturer's instructions, then reverse‐transcribed followed by polymerase chain reaction (PCR) of the ALK kinase domain by nested PCR (first primer: F, AGCCCTGAGTACAAGCTGAGC, R, CCATATTCTATCGGCAAAGCGGT; second primer: F, TCAGTGACCTGAAGGAGGTGC, R, TGTTGATTACATCCGGGTCCTGG). PCR products were sequenced using ABI PRISM 3130x Genetic Analyzer (Thermo Fischer Scientific, Waltham, MA, USA). Sequencing data were analyzed with ApE‐A plasmid Editor v2.0.5 and the National Center for Biotechnology Information Basic Local Alignment Search Tool.

### Next generation sequencing

Genomic DNA was extracted with a DNeasy Blood and Tissue Kit (Qiagen, Hilden, Germany). A library was prepared using a HaloPlex custom panel (Agilent Technologies, Inc., Santa Clara, CA, USA), which is designed to detect well‐known cancer‐associated somatic mutations.^15^ The library was then purified and quantified using quantitative PCR, sequenced on Illumina MiSeq Reagent Kit v2 (300 cycles). The sequence reads were aligned to the University of California Santa Cruz hg38 reference genome using HISAT2 (version 2.1.0). Read pairs with a mapping quality of <30 were excluded.

### Cell lines and reagents

Ba/F3 immortalized murine bone marrow‐derived pro‐B cells were cultured with Dulbecco's modified Eagle medium (DMEM) low glucose supplemented with 10% fetal bovine serum (FBS) and kanamycin (250 mg/mL; Meiji Seika Pharma, Tokyo, Japan) and 0.5 ng/mL of interleukin‐3 (IL‐3). Human kidney embryo cell lines, 293FT cells were cultured in DMEM high glucose with 10% FBS. The cells were infected with lentivirus replicated in 293FT cells by transforming them with paging plasmids (Virapower), which express rearranged cDNA regions encoding EML4‐ALK variant 1 and either WT or different mutations (I1171S and I1171S + G1269A). Cells were selected with blasticidin (7 μg/mL) for one week. After selection, IL‐3 was withdrawn from the culture medium.

Crizotinib (PF‐02341066), lorlatinib (PF‐06463922), and brigatinib (AP26113) were obtained from Shanghai Biochempartner (Shanghai, China). Alectinib (CH5424802), and ceritinib (LDK‐378) was purchased from ActiveBiochem (Hong Kong).

### Antibodies and immunoblotting

Ba/F3 cells (1 × 10^6^) were seeded into 12‐well plates and treated with different drugs at different concentrations for three hours. Cells were suspended in lysis buffer containing 0.1 M Tris (pH 7.5), 10% glycerol and 1% sodium dodecyl sulfate (SDS), and boiled at 100°C for five minutes. The protein concentrations were measured with a BCA protein assay Kit (Thermo Fischer Scientific). The lysates were adjusted to 0.9 μg/μL with lysis buffer and 20% volume of the sample buffer containing 0.65 M Tris (pH 6.8), 20% 2‐mercaptoethanol, 10% glycerol, 3% SDS, and 0.01% bromophenol blue was added. Next, 10 μg of each sample was electrophoresed using SDS‐polyacrylamide gel electrophoresis (SDS‐PAGE) and immunoblotted with the antibodies against total‐ALK (#3633S Cell Signaling Technology), phospho‐ALK (#3341 Cell Signaling Technology), and glyceraldehyde 3‐phosphate dehydrogenase (GAPDH) (MAB374; Millipore, Billerica, MA, USA).

### Cell viability assays

The cells were seeded into 96‐well plates and treated with serial dilutions of different inhibitors. Each condition was performed in triplicate. For Ba/F3 cells, 2000 cells were plated into 96‐well plates and cultured in the medium containing different drugs for 72 hours. After incubation, the cells were incubated with CellTiter‐Glo reagent (Promega) for 10 minutes, and luminescence was measured using TriStar LB941 (Berthold Technologies, Bad Wildbad, Germany). GraphPad Prism version 8.0 (GraphPad Software Inc., San Diego, CA, USA) was used to graphically display the data. The half‐maximal inhibitory concentration (IC_50_) values were determined using a nonlinear regression model with a sigmoidal dose response in GraphPad.

## Results

**Figure 1:**
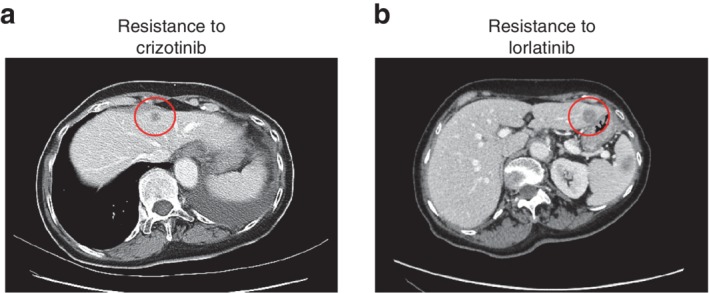
Computed tomography (CT) image of the case. (**a**) Low‐density area (LDA) in liver is found and biopsy was performed, but no cancer cells were detected. (**b**) CT image after lorlatinib treatment shows new LDA in the liver left lobe.

The patient in this report was a 68‐year‐old woman who was diagnosed with stage III NSCLC with ALK rearrangement in March 2011. The patient initially underwent left pneumonectomy and was followed‐up for one year. After follow‐up, her computed tomography (CT) revealed liver and spleen metastasis, therefore chemotherapy was started with cisplatin/pemetrexed/bevacizumab for four cycles. In December 2012, her CT scan revealed recurrence of spleen metastasis, so chemotherapy was discontinued and she was switched to crizotinib therapy. The tumor shrank, but in 2013 liver metastasis appeared, and liver biopsy was performed but failed to include tumor cells (Fig 1a). Alectinib treatment was initiated, but her tumor continued to grow. The patient was then enrolled in a study that included lorlatinib treatment. After two years, her CT scan showed another liver metastasis progression, for which the patient underwent a second liver metastasis biopsy (JFCR 49‐2) (Fig 1b).

**Figure 2:**
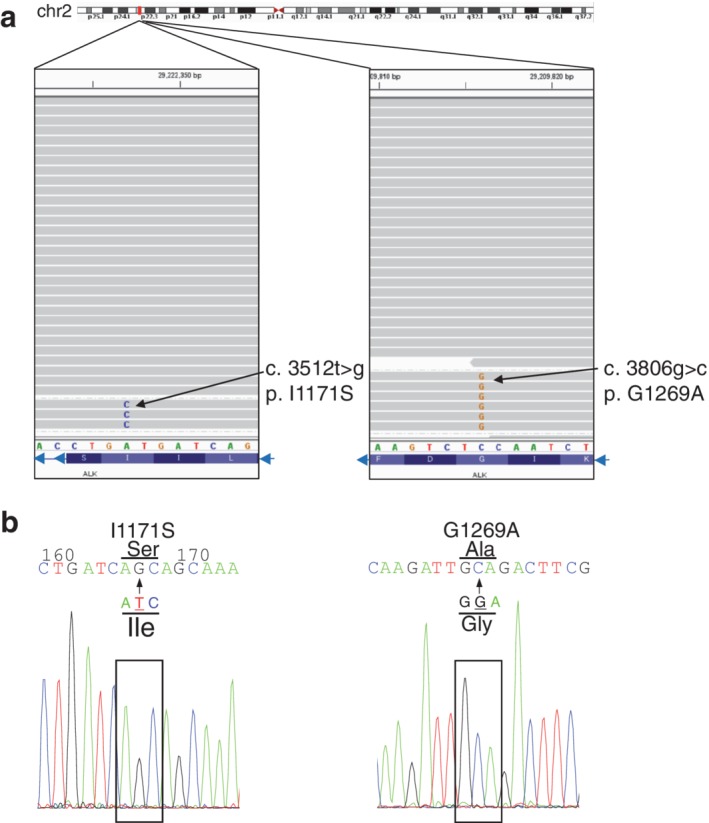
Sequencing data from the next‐generation sequencing (NGS) and Sanger sequencing. (**a**) Next‐generation sequencing shows nucleotide 3512 t > g mutation and 3806 g > c mutation. (**b**) cDNA from the total RNA of the liver biopsy sample was used to amplify ALK by PCR, and Sanger sequencing of the ALK kinase domain showed nucleotide 3512 t > g mutation and 3806 g > c mutation as major peak.

**Figure 3:**
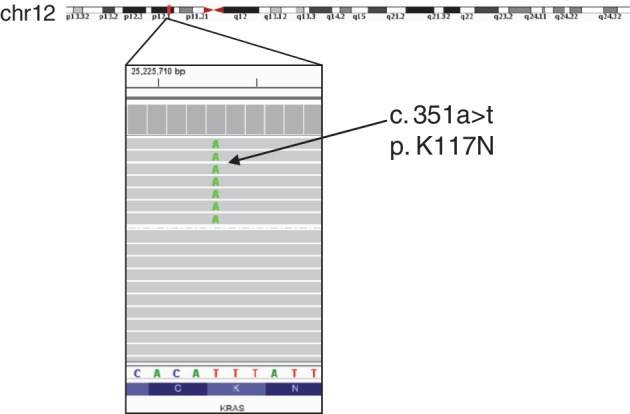
Sequencing data of next generation sequencing from JFCR‐49‐2 revealed KRAS K117N mutation.

We performed next‐generation sequencing (NGS) on the liver biopsy tissue and it showed ALK I1171S and G1269A mutation reads (Fig 2a). Because of the low tumor cellularity in the obtained liver biopsy sample, the number of reads in which mutation is confirmed in NGS represented only about 2% of the reads. In addition to ALK compound mutation, our NGS analysis also revealed the KRAS K117N mutant with a few read counts which is also reported as a resistance mechanism of ALK inhibitor (Fig 3).^16^, ^17^ We tried to identify ALK mutation from expressing ALK fusion protein in the same sample. Therefore, reverse‐transcribed cDNAs from the total RNA of the sample were used to amplify ALK by PCR, and we checked the sequence of the ALK kinase domain using Sanger sequencing. The results showed that 3512 t > g and 3806 g > c mutations in *ALK* were detected as the major peak (Fig 2b). Based on these facts, it was suggested that JFCR 49‐2 acquired resistance to lorlatinib because it has compound mutation I1171S and G1269A *in cis*. We prepared Ba/F3 cells with overexpressing EML4‐ALK WT and I1171S single mutation and I1171S + G1269A double mutation. Of note, EML4‐ALK with I1171S + G1269A mutation harboring Ba/F3 cell line grew without IL‐3, suggesting that this mutant EML4‐ALK does not lose oncogenicity.

**Figure 4:**
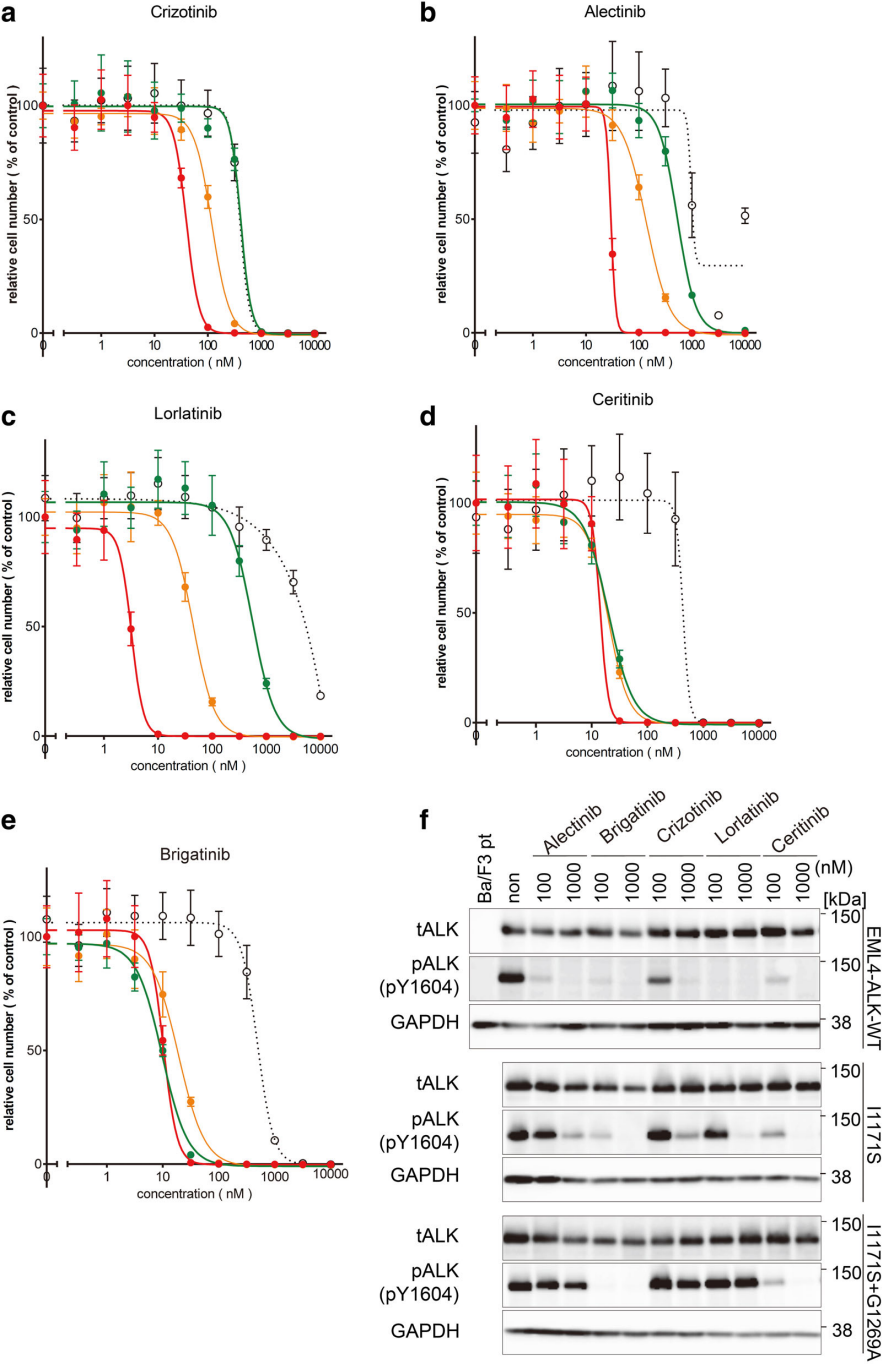
Ceritinib and brigatinib overcome Ba/F3 cells with I1171S + G1269A double mutation. (**a**–**e**) Ba/F3 EML4‐ALK WT cells, I1171S cells, and I1171S + G1269A cells were treated with the indicated concentration of crizotinib, alectinib, lorlatinib, ceritinib and brigatinib. Cell viability was analyzed using the CellTiter‐Glo assay. 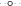
Ba/F3, 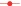
EML4‐ALK (WT), 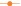
I1171S, 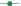
I1171S+G1269A. (**f**) Immunoblotting analysis of phosphor‐ALK in EML4‐ALK‐WT cells, I1171S cells, and Ba/F3 I1171S + G1269A cells. The cells were treated with indicated concentrations of ALK ‐TKIs. ALK phosphorylation in the compound mutation harboring cells was suppressed in ceritinib and brigatinib.

The cell viability assay using CellTiter‐Glo Kit was performed with five different ALK inhibitors: crizotinib, alectinib, ceritinib, lorlatinib and brigatinib. In crizotinib, alectinib, and lorlatinib treatment, it was shown that the sensitivity for the drugs was decreased in the order of EML4‐ALK‐WT, I1171S single, and the compound mutation. The IC_50_ values of alectinib, crizotinib and lorlatinib for I1171S + G1269A mutant cells were 412.7, 531.4, and 552.6 nM, respectively (Fig 4a–c), suggesting that these drugs are thought to be ineffective against compound mutantion in the clinical case. In contrast, ceritinib and brigatinib have very low IC_50_ values (ceritinib, 20.0 nM; brigatinib, 9.74 nM) (Fig 4d,e), and these inhibitors are also similarly effective against EML4‐ALK‐WT and I1171S single mutant‐expressing cells. Moreover, immunoblotting analysis showed that ALK phosphorylation was suppressed in brigatinib and ceritinib at lower doses (Fig 4f). From these results, it follows that if I1171S and G1269A compound mutation is observed, the resistance would be overcome with ceritinib or brigatinib.

Indeed, ceritinib at a dose of 750 mg/day was selected for subsequent molecular targeted therapy, and it inhibited liver metastasis progression for four months. However, because of grade 2–3 gastrointestinal symptoms such as anorexia and vomiting, dose reduction was necessary, and eventually treatment was discontinued.

## Discussion

Since ALK fusion gene positive lung cancer was first reported in 2007,^18^ various ALK‐TKIs have been developed and clinically used in various countries all over the world. Although high response rates of the ALK‐TKIs have been reported, acquired resistance mechanisms have also been noted such as gatekeeper mutation, ALK fusion gene amplification, and activation of alternative signaling pathways such as epidermal growth factor receptor (EGFR), c‐Kit, and KRAS.^19^, ^20^ The current treatment strategy is sequential ALK targeted therapy regardless of what mutant was acquired by first‐line ALK inhibitor treatment. Resistance against the third‐generation ALK‐TKI lorlatinib, which is currently the later line of therapy, has been reported in multiple different compound mutations found via mutagenesis screening and clinical samples.^7^, ^8^ In fact, in one patient, different resistance mutation harboring cells were simultaneously detected. The occurrence of multiple compound mutations is attributed to the diversity of cancer cells in the tumor tissue. In this study, our NGS results showed a few read counts of KRAS K117N mutation in addition to ALK compound mutation. This suggests that various responses to molecular targeted therapy could have occurred, and it also reflects intratumor heterogeneity. Therefore, the treatment strategy of ALK‐TKI will become diverse and difficult. Because of the mixed/complex resistant mechanisms, there may be limitations to use different kinds of ALK‐TKI. In some instances, chemotherapy plus immune checkpoint inhibitor might be a reasonable alternative.

In this study, NGS analysis was performed using a biopsy specimen of liver metastasis after resistance to lorlatinib treatment, and ALK I1171S and G1269A compound mutation was found. Although the distance between I1171S and G1269A mutations is 98 amino acids, a library for this NGS was prepared from genomic DNA that contains long introns between these two mutations, and even if we used cDNA for detection of these mutations, the length of the sequencing reads is too short (approximately 150 bp) to find these two mutations in the same reads. A different technique such as long‐read sequence would be required to confirm if the mutations were* in cis* or *in trans*. In our study, we could fortunately confirm that mutations were *in cis* by amplifying ALK fusion gene from cDNA of the biopsy tissue. But if the option of detecting the mutations from cell‐free DNA in blood was considered then it would be impossible to clarify if the distant mutations were *in cis* or *in trans*.

No tumor cells were found in the liver metastasis biopsy after crizotinib resistance, so it is unclear which of the mutations I1171S and G1269A was acquired first. In this clinical course, NGS of liquid biopsy was also performed after lorlatinib resistance, and mutations of ALK I1171S and G1269A were independently observed. Liquid biopsy NGS showed that G1269A was approximately twice as many as the I1171S read count. This fact may suggest that I1171S single mutant was first acquired after crizotinib treatment, and sequential ALK inhibitor treatment decreased I1171S single mutation and the acquisition of new G1269A mutation after lorlatinib caused compound mutation. This speculation is consistent with the ineffectiveness of alectinib after crizotinib, as I1171S mutant ALK is known to be resistant to alectinib.

Treatment of ALK rearranged positive lung cancer requires sequential use of first‐, second‐, and third‐generation ALK inhibitors, but the sensitivity of these drugs differs for each resistance, and individualized treatment will be required for patients. Analysis of acquired mutations for each drug and treatment strategies will be essential, and multiple analyses along the clinical course progression similar to that reported in this study might be required promptly for a better prognosis of patients with ALK‐rearranged NSCLC.

## Disclosure

R. Katayama reports research grants from Chugai, TAKEDA, TOPPAN Printing, FUJIFILM, Daiichi‐sankyo, Taiho, and lecture fees from Pfizer, outside the submitted work. M. Nishio reports grants and personal fees from Ono Pharmaceutical, grants and personal fees from Bristol Myers Squibb, grants and personal fees from Pfizer, grants and personal fees from Chugai Pharmaceutical, grants and personal fees from Eli Lilly, grants and personal fees from Taiho Pharmaceutical, grants and personal fees from AstraZeneca, personal fees from Boehringer‐Ingelheim, grants and personal fees from MSD, grants and personal fees from Novartis, personal fees from Sankyo Healthcare, personal fees from Taiho Pharmaceutical, personal fees from Merck Serono, grants from Astellas, outside the submitted work. N. Fujita reports grants from Api Co., Ltd., outside the submitted work. There are no further disclosures related to this work.
